# Detection of enterotoxigenic *Staphylococcus aureus* isolates in domestic dairy products

**Published:** 2010-09

**Authors:** AA Imani Fooladi, HR Tavakoli, A Naderi

**Affiliations:** 1 Research Center of Molecular Biology; 2 Health Research Center, Baqiyatallah University of Medical Sciences Theran, Iran; 3 Department of Biological Sciences, Alzahra university, Theran, Iran

**Keywords:** *Staphylococcus aureus*, Enterotoxin, Food poisoning, Dairy products

## Abstract

**Background and objectives:**

*Staphylococcus aureus*is a one of THE most frequent causes of food poisoning (FP) in dairy products. The main etiologic agents of FP are staphylococcal enterotoxins (SE). There are different types of SE; types A (SEA) and B (SEB) are the most clinically important enterotoxins. Traditional dairy products are still produced in small batches and sold by some vendors without a permit from the Ministry of Health. This study focuses on the molecular and serological detection of enterotoxigenic *Staphylococcus aureus* SEA and SEB genes and its products, respectively from samples of such traditional products.

**Materials and Methods:**

100 samples from dairy products were produced under sterile conditions via traditional methods and were transported to the laboratory. The samples were cultured and identified by routine bacteriological methods. The isolated bacteria were evaluated by PCR tests for detection of the genes encoding SEA and SEB. Subsequently, the ability of these strains to produce enterotoxin was examined by Sac's culture method and was confirmed by Sigel Radial Immounodiffussion (SRID).

**Results:**

The results indicated that 32% of the dairy products were contaminated by *S. aureus* (cream 18%, cheese 10%, milk 4%). The PCR results showed that 15.6% of the *S. aureus* isolates possessed the SEA gene, 9.3% had the SEB gene, and 6.2% possessed both genes. The evaluation of enterotoxin production indicated that 80% of SEA and 33% of SEB genes were expressed.

**Conclusion:**

Enterotoxins SEA and SEB are heat stable and consequently; heating has no effect on dairy products contaminated by entertoxins. Subsequently, gastritis may occur within several hours after consumption. Our findings suggest that PCR is a rapid, sensitive, specific, and inexpensive method for detecting SE and can replace the traditional assays.

## INTRODUCTION

*Staphylococcus aureus* Food Poisoning (FP) is a common cause of food-borne disease worldwide (1). Classically, enterotoxins from *Staphylococcus aureus* strains can be classified into 18 serological types: A-U (except S, F and T) (2). Most enterotoxin serotypes are heat stable and may resist inactivation by gastrointestinal proteases like pepsin. The B and C serotypes are cleaved by digestive enzymes in the cysteine loop site, but this cleavage is not effective against their toxicity and antigenic properties (1, 3) (4). Staphylococcal enterotoxin (SE) A and SEB are two of the most important gastroenteritis causing agents. In some areas, more than 50% of FP is caused by SEA. SEA and SEB are the most FP agents (>60%) in USA and England (5).

*Staphylococcus aureus* nasal carriage is established constantly in 20%–40% of healthy human population and intermittently in 60% and only 10%–20% of people are non-carriers (6). If food providers don't abide by the rules of hygiene, they can transfer the contamination to food. A concentration of 105 bacteria/gram in foods is sufficient for toxin production and induction of disease (1, 5).

Staphylococcal enterotoxins are low molecular weight proteins (MW 26.900-29.600 KD). These are encoded by genes embedded in mobile genetic elements such as phages, (not in plasmids) and pathogenicity islands (7). Heat resistance is one of their most important physical and chemical properties; their biological activity remains unchanged even after thermal processing of food (7–9). For the above mentioned reason, these toxins can cause epidemic gastroenteritis. Actually, SEB is the most important enterotoxin that causes gastroenteritis.

Several studies have shown that 15% to 80% of the *S. aureus* isolated from various sources (dairy products, ice cream, meat products …) are able to produce enterotoxin (10–12). There are several methods for detection of enterotoxigenic bacteria. The phenotypical methods (agglutination, SRID) are not reliable in specificity, because SE serotypes are antigenically similar (13). On the other hand, commercial serologic kits can not detect all the serotypes and is limited to serotypes (A-E) (14). Therefore, molecular techniques such as PCR and real-time- PCR are recommended for detection of *S. aureus* enterotoxin genes. However, a gene's presence does not establish its enterotoxigenic properties of a strain. Therefore, the expression of the gene should also be evaluated (14). In this study, both genotypic and phenotypic methods were utilized to detect SEA and SEB genes and its products. Furthermore, we used these methods to examine the contamination rates of traditional dairy products by enterotoxigenic (SEA and SEB) *S. aureus*.

## MATERIALS AND METHODS

**Dairy specimen collection and screening.** From every dairy samples (n = 100), 100 grams of milk, cream or cheese were collected randomly from dairy products sellers in 4 areas of Tehran. Depending on type (solid or liquid), each sample was diluted 1/100 in saline (15). From each solution produced, 1ml was transferred to Cook meat media culture with 9% NaCl and incubated at 37°C for 48h. In the second phase, 0.1 ml from each previously cultured medium was transferred to Baird-Parker agar and nutrient agar (Sigma Co) and incubated for 24–48 h. Estimates of colony numbers were obtained by counting with colony counters. If black colonies with transparent zone were produced in Baird-Parker, confirmation tests (gram staining, coagulase, catalase, DNase and manitol salt agar) were carried out for final identification of *S. aureus* (6, 16).

**Fig. 1:**
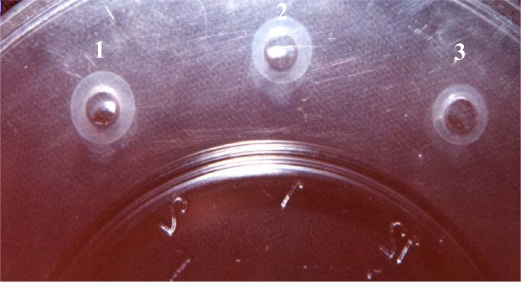
SEA & SEB types were determined by the SRID method. 1. Reaction between Anti SEA and SEA standard 2. Reaction between Anti SEB and SEB standard 3. Reaction between Anti SEB and produced SEB (control test).

**Study of SEs production ability by phenotypical methods.** The ability of strains to produce enterotoxin was examined by Sac culture methods (15). Total protein was measured using the Bradford method and confirmed with SDS-PAGE electrophoresis. SEA & SEB types were determined with the SRID method after crude metabolite concentration by sucrose gradient (17–19). (Fig. 1).

**PCR experiments.** The genomic DNA from every isolates of *S. aureus* strain was extracted by the modified phenol-chloroform method. Lysates of colonies were prepared according to instruction given by Sharma *et al.* (20).

The sequences and corresponding sequence locations of the oligonucleotide primers used in this study are shown in Table 1. One forward primer common two enterotoxin genes and two reverse primers were used (20) (Table 1). PCR reactions were performed in reaction buffer (10x), MgCl_2_ (4 mM) in a total volume of 50 µl, containing 1 µl (∼1ng) of template DNA, 20–30 pM each of the primers SA-U, SA-A for SEA, and SA-U, SA-B for SEB, separately, 0.2 mM of mixed deoxynucleotide tri-phosphates, and 1 unit of Taq DNA polymerase. PCR was performed under the following conditions: initial denaturation at 94°C for 4 min, subsequently followed by 35 cycles of 94°C for 30 s, 50°C for 30 s, and 72°C for 30 s with a final extension of 10 min at 72°C (26). We used SEB positive strain (*S. aureus* Col1) and SEA positive strain (*S. aureus* HN2) as positive reaction control strains.

**Table 1:** Details of primers and amplicons

Primer name and size	Description	Nucleotide sequence	Gene location	PCR product size
SA-U (20)	Universal forward primer	5-TGTATGTATGGAGGTGTAAC-3	–	
SA-A (18)	Reverse primer for *sea*	5-ATTAACCGAAGGTTCTGT-3	639–657	270
SA-B (18)	Reverse primer for *seb*	5-ATAGTGACGAGTTAGGTA-3	564–582	165

**Fig. 2:**
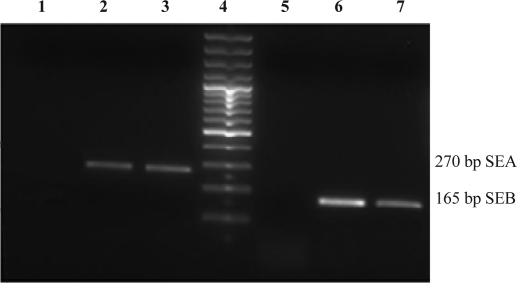
Agarose gel electrophoresis patterns showing PCR amplification. The individual toxin gene products were characterized by comparing them with standard molecular size markers. Lanes 1 Neg control, Lanes 2 experiment sample and Lanes 3 Positive control of SEA (270 bp); Lane 4, 100 bp size marker; Lane 5 negative control for SEB; Lanes 6 experiment sample and 7 positive control SEB (165 bp).

A 10 µl aliquot of the amplified PCR product was analyzed on 2% TAE agarose gel containing 0.5 µg/ml ethidium bromide. Electrophoresis was performed at 80V for 1 h. Gels were viewed by UV transillumination and photographed (Fig. 2).

**Statistical Analysis.** Statistical analyses were performed using the chi-square test All statistical analyses were conducted using SPSS 13.0 software (SPSS Inc., Chicago, IL).

## RESULTS

**The frequency of contaminated dairy products.** We determined 32 *S. aureus* from food samples: 18 from cream, 10 from cheese, and 4 from milk (Table 2). We detected each of the toxin gene (SEA, SEB) products by multiplex PCR and compared the results with the phenotypic method. The most frequently contaminated sample by *S. aureus* was cream (18%), and the least frequently contaminated was milk (4%). Overall, 32% of the samples were contaminated. The numbers of bacteria in contaminated samples by CFU/ml varied (Table 2 and Fig. 4).

**Table 2:** SEs production ability evaluated by a phenotypical ( traditionally) method and detection of SEs gene by genotypical (PCR) method. 31.1% of all isolated *S. aureus* from dairy products had one or both the SEA & SEB genes but only 15.5% of *S. aureus* isolated from dairy products was enterotoxinogenic.

					***Enterotoxins***				
**Type of food sample**	**Place No**	**Sample Size (n)**	**No of Isolates (n)**	**Genotypic method(No of positive)**			**Phenotypic method(No of positive)**		

				**n**	**%**	**Type**	**n**	**%**	**Type (n)**

	1	10	6	2	33.3	SEA, SEB	1		
Cream	2	9	5	–	–	–	–	16.6	
	3	8	4	1	25	SEA	–	–	–
	4	8	3	2	66.6	SEA SEB+SEA	1	–	–
						SEA		33.3	SEA
									
	1				33.3				
	2	10	3	1	–	SEA	1	33.3	SEA
Cheese	3	8	2	–	100	–	–	–	–
	4	9	1	1	25	SEB	1	100	SEB
		8	4	1		SEA+SEB	–	–	–
									
	1								
	2	8	1	1	100	SEA	1	100	SEA
Milk	3	7	1	–	–	–	–	–	–
	4	8	2	1	50	SEB	–	–	–
		7	–	–	–	–	–	–	–
Total	4	100	32	10	31.1		5	15.5	

**SEs production ability by phenotypical method.** The results showed that 15.6% of the S.aureus strains isolated from dairy products were enterotoxinogenic. (12.5% SEA and 3.1% SEB) The maximum numbers of enterotoxin producer isolates were detected in cream, where 2 (6.2%) isolates of *S. aureus* were positive for SEA and SEB production of enterotoxin. The enterotoxin B (SEB) was produced only in one isolate (3.1%) of *S. aureus* from cheese. No isolates were able to produce both enterotoxins simultaneously (Tables 2 and 3).

**Table 3:** Prevalence of staphylococcal enterotoxin A, B in *S. aureus* isolates from food samples evaluated by the genotypic method and phenotypic method.

		No. of enterotoxin positive samples							
	
Origin of *S.aureua*	No. of tested	Genotypic method				Phenotypic method			
	
		**SEA**	**SEB**	**SEA + SEB**	**%**	**SEA**	**SEB**	**SEA+SEB**	**%**
Cream	18	3	1	1	15.6	2	–	–	6.2
Cheese	10	1	1	1	9.3	1	1	–	6.2
Milk	4	1	1	–	6.2	1	–	–	3.1
Total (n)%	32 (100%)	5	3	2	31.1	4	1	–	15.5

**Detection of SEs gene by genotypical method.** The studies revealed that 31.1% all the *S.aureus* isolated from dairy products had one or both of theSEA and SEB genes (15.6% SEA, 9.3% SEB and 6.2% both). The maximum number of enterotoxin-positive isolates came from cream, where 5(15.6%) isolates of *S. aureus* were positive for SEA and SEB enterotoxin genes (Tables 2 and 3).

## DISCUSSION

The existence of *S. aureus* in foods and dairy products was confirmed in the 19th century (19). In 1941, Barber described the symptoms of food poisoning, resulting from *S. aureus* contamination (21). The variation of staphylococcal isolates in their ability to produce enterotoxins depend on the source (21). Since human interference, the diary food producers, determines the level of contamination of dairy products. Our result shows that milk was less contaminated than cream, because the more handling, the more contamination. In the current study, 32% of non clinical isolates possessed SEA & SEB genes, but only15.6% of them were enterotoxigenic. We were able to evaluate the enterotoxin genes genotypically. However, we examined bovine milk dairy products for which there was no information about health condition of producer animals. According to Harvi and Gilmo studies, 3.9% to 6% of isolated *S. aureus* from safe bovine milk produces enterotoxin (16). Our results disagree; this is not surprising because only phenotypical studies were carried out in their study and the sources of diary product, preparation methods, and hygiene standard were probably different (16). One of the most important problems is that *S. aureus* can be responsible for food poisoning by enterotoxin production (23). In agreement with another study, we found that 32% of all dairy products were contaminated by *S.aureus* (22). Obviously, the quantity of *S. aureus* in food products are related to many factors: the number of contaminated carriers and personnel in preparing the food, ignoring the rules of hygiene in food factories, transport systems, and rate of animal contamination. All of the factors need to be controlled separately. Future studies should examine dairy products that are produced under sterile conditions and compare them with rural dairy products.

**Fig. 3:**
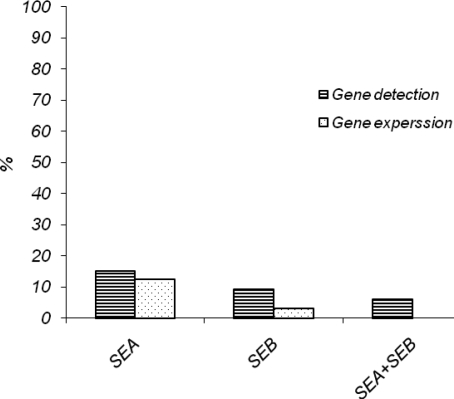
The frequency of SEA and SEB genes existence and gene expression.

In the current study, after isolation of *S. aureus* from dairy products, the enterotoxin genes were detected by PCR technique. The results revealed that 31.1% of isolates have one or both of the SEA and SEB genes. The genotypic and phenotypic detection methods are compared in Tables 1 and 2 and Fig. 3. These results expose the lower degree of sensitivity of the phenotypical method. However, a possible limitation of this procedure is that the molecular methods are only able to demonstrate the existence of the genes encoding for SEs in bacteria but cannot prove that production of SEs protein occurs unless RT-PCR is carried out (15). High percentages of *S. aureus* with enterotoxin genes, especially SEA, were confirmed phenotypically and the results of PCR showed a clear relationship with immune assay results, in agreement with our previous study and those of others (8, 24, 25). Nevertheless, with regard to SEB, more differences are observed between genotypical and phenotypical methods. Under the best conditions, 40% to 50% of isolates with the SEB gene are capable of enterotoxin production (26). This is in agreement with our results. Therefore, the expression of enterotoxin genes depends on factors such as the origin and identity of the bacterial isolate and the host environment of bacteria. The host plays an important role in assisting an adaptation between the bacteria and their surrounding environment. For example most of the bacteria isolated from cows produce SEA & SED (15, 27) while bacteria isolated from goats and sheep produce SEC (27) and most bacteria isolated from skin and human wounds produce SEB (28). Moreover, the results showed that the phenotypical methods are less sensitive. The techniques based on DNA are able to determine the presence of enterotoxin genes but cannot demonstrate their expression. The relation between the presence of the genes in bacteria and the rates of expression can be determined with serological and immunological tests.

**Fig. 4:**
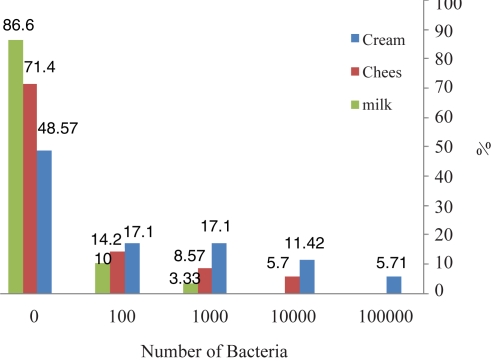
The frequency of contaminated dairy products. The samples most commonly contaminated by *S. aureus* were cream, and the least contaminated were milk.

## References

[1] Paciorek ML, Kochman M, Piekarska K, Grochowska A, Windyg B (2007). The distribution of enterotoxin and enterotoxin-like genes in *Staphylococcus aureus* strains isolated from nasal carriers and food samples. Int J of Food Microbiol.

[2] Holtfreter S, Bröker BM (2005). Staphylococcal superantigens: Do they play a role in sepsis?. Arch. Immunol. Ther. Exp.

[3] Heng C, Merlin SB (1979). Purification and some physichemical 3. properties of staphylococcus enterotoxin D. Ame. Chem. Society.

[4] Rossalxn D (1990). B-. Large scale purification of staphlococcal 4. Entertoxins A, B and C_2_ by dye ligand affinity chromatography. Appl and Enviro micro.

[5] Kluytmans JAJW, Wertheim HFL (2005). Nasal carriage of Staphylococcus aureus and prevention of nosocomial infections. Infection.

[6] Ruimy R, Angebault C, Djossou F, Dupont C, Epelboin L, Jarraud S Are Host Genetics the Predominant Determinant of Persistent Nasal Staphylococcus aureus Carriage in Humans?. J Infect Dis.

[7] Martin MC, Fueyo JM, Gonzales-Hevia JM, Mendoza MC (2004). Genetic procedure for identification of enterotoxigenic strains of *Staphylococcus aureus* from three food poisoning outbreaks. J Food Microbiol.

[8] McLauchlin J, Narayanan GL, Mithani V, O'Neill G (2000). The detection of enterotoxins and toxic shock syndrome toxin genes in *Staphylococcus aureus* by polymerase chain reaction. J Food Prot.

[9] Chapaval L, Moon DH, Gomes E, Duarte F R, Tsai S M (2006). Use of PCR to detect classical enterotoxin genes and toxic shock syndrome toxin-1 gene in *Staphylococcus aureus* isolated from crude milk and determination of toxin productivities of *S. aureus* isolates harboring these genes. J Arq Inst biol.

[10] Omoe K, Hu DL, Takahashi-Omoe H, Nakane A, Shinagawa K (2005). Comprehensive analysis of classical and newly described staphylococcal superantigenic toxin genes in *Staphylococcus aureus* isolates. FEMS Microbiol Lett.

[11] Fueyo JM, Mendoza MC, Alvarez MA, Martin MC (2005). Relationships between toxin gene content and genetic background in nasal carried isolates of *Staphylococcus aureus* from Asturias, Spain. FEMS Microbiol Lett.

[12] Bania J, Dabrowska A, Korzekwa K, Zarczynska A, Bystron J, Chrzanowska J, Molenda J (2006). The profiles of enterotoxin genes in *Staphylococcus aureus* from nasal carriers. Lett. Appl. Microbiol.

[13] Edwin C, Tatini SR, Maheswaran SK (1986). Specificity 1 and crossreactivity of staphylococcal enterotoxin A monoclonal antibodies with enterotoxins B, C1, D, and E. Appl. Environ Microbiol.

[14] Van Belkum A (2003). Molecular diagnostics in medical microbiology: Yesterday, today and tomorrow. Curr. Opin Pharmacol.

[15] Morandi S, Brasca M, Lodi R, Cremonesi P, Castiglioni B (2007). Detection of classical enterotoxins and identification of enterotoxin genes in *Staphylococcus aureus* from milk and dairy products. Veter Microbiol.

[16] Gilmour A, Harvey J (1990). staphylococci in milk and milk Product. J of Appl Microbiology.

[17] Schantz EJ (1972). Purification and some chemical properties of staphylococcal Enterotoxin. A Biochemistry.

[18] Bradford M M (1976). A rapid and sensitive method for the Quantitation of microgarm Quantities of protein utilizing the principle of protein – dye binding. Analytical biochemistry.

[19] Abbar M Selected biological properties of enterotoxigenic staphylococci isolated from milk.

[20] Sharma NK, Rees CED, Dodd CE (2000). Development of a single-reaction multiplex PCR toxin typing assay for *Staphylococcus aureus* strains. Applied and Enviro Microbiol.

[21] Bennet RW (1986). *Staphylococcus aureus* identification characteristics and enterotoxigenicity. J of Food science.

[22] Casman EPRW, Bennet AE (1967). Identification of a fourth staphylococcal enterotoxin D. J of Bacteriol.

[23] Bradley GS, Teresa K (2005). Staphylococcal enterotoxins: A purring experience in review, Part I. Clin microbiol News Letter.

[24] Anvari SH, Sattari M, Forozandehe Moghadam M (2008). Najar Peerayeh SH,Imanee Fouladi AA. Detection of *Staphylococcus aureus* Enterotoxins A to E from clinical samples by PCR. Res J of Biolo Scie.

[25] Jorgensen HJ, Mork T, Hogasen HR, Rovik LM (2005). Enterotoxigenic *Staphylococcus aureus* in bulk milk in Norway. J Appl Microbiol.

[26] Najera-Sanchez G, Maldonado-Rodriguez R, Ruiz Olvera P, Dela Garza L M (2003). Development of two multiplex polymerase chain reactions for the detection of enterotoxigenic strains of *Staphylococcus aureus* isolated from foods. J Food Protocol.

[27] Normanno G, Firinu A, Virgilio S, Mula G, Dambro-sio A, Poggiu A, Decastelli L (2005). Coagulase-positive staphylococci and *Staphylococcus aureus* in food products marketed in Italy. Int J Food Microbiol.

[28] Imanifooladi A A, Sattari M, Najar Peerayeh SH, Hassan Z M, Hossainidoust S R (2007). Detection the *Staphylococcus aureus* producing enterotoxin isolated from skin infection in hospitalized patients. Pak.J.Bio.Scie.

